# Pre-conditioning of Equine Bone Marrow-Derived Mesenchymal Stromal Cells Increases Their Immunomodulatory Capacity

**DOI:** 10.3389/fvets.2020.00318

**Published:** 2020-06-11

**Authors:** Valeria Caffi, Gabriel Espinosa, Gonzalo Gajardo, Natalia Morales, María Carolina Durán, Benjamín Uberti, Gabriel Morán, Anita Plaza, Claudio Henríquez

**Affiliations:** ^1^Instituto de Farmacología y Morfofisiología, Facultad de Ciencias Veterinarias, Universidad Austral de Chile, Valdivia, Chile; ^2^Escuela de Graduados, Facultad de Ciencias Veterinarias, Universidad Austral de Chile, Valdivia, Chile; ^3^Instituto de Ciencias Clínicas, Facultad de Ciencias Veterinarias, Universidad Austral de Chile, Valdivia, Chile; ^4^Instituto de Medicina, Facultad de Medicina, Universidad Austral de Chile, Valdivia, Chile

**Keywords:** horse, mesenchymal stromal cells, lymphocytes, proliferation, immunomodulation

## Abstract

Mesenchymal stem/stromal cells (MSCs) are increasingly explored for the treatment of degenerative and inflammatory diseases in human and veterinary medicine. One of the key characteristics of MSCs is that they modulate inflammation mainly through the secretion of soluble mediators. However, despite widespread clinical use, knowledge regarding the effector mechanisms of equine MSCs, and consequently their effectiveness in the treatment of diseases, is still unknown. The objectives of this study were to determine the mechanisms underlying inhibition of lymphocyte proliferation by equine bone marrow-derived MSCs, and to evaluate the effect of pre-conditioning of equine MSCs with different pro-inflammatory cytokines on inhibition of lymphocyte proliferation. We determined that inhibition of lymphocyte proliferation by equine MSCs depends on activity of prostaglandin-endoperoxide synthase 2 and indoleamine 2,3-dioxygenase. Additionally, pre-conditioning of MSCs with TNF-α, IFN-γ or their combination significantly increased the expression of prostaglandin-endoperoxide synthase 2, indoleamine 2,3-dioxygenase, iNOS and IL-6. This upregulation correlated with an increased inhibitory effect of MSCs on lymphocyte proliferation. In conclusion, pre-conditioning of bone marrow-derived MSC increases their inhibitory effect on lymphocyte proliferation in horses.

## Introduction

Multipotent mesenchymal stem/stromal cells (MSCs) are a heterogeneous fibroblastic-like subset of cells originally obtained from the stromal compartment of bone marrow (BM) (1, 2). Currently, MSCs can be obtained from a number of tissues, such as adipose tissue, umbilical cord blood, peripheral blood, dental pulp, placenta, and other solid mesenchymal tissues (3–7). MSCs in all species are characterized by their adherence to plastic in cell culture, expression of surface markers and their potential differentiation into three mesodermal lineages: chondrocytes, osteoblasts and adipocytes (8). This allows MSCs to be involved in tissue reparation and regeneration of injured tissue (9, 10). In addition, MSCs possess strong immunomodulatory properties which are mediated by cell-cell contact and through the production of a plethora of soluble factors (11, 12). MSCs are able to modulate different components of the immune response, either of the innate or adaptive system, apparently without compromising the response against pathogens (13–15). Another important feature of MSCs is they are hypoimmunogenic and can be safely administered to allogeneic hosts (16).

Neutrophils are some of the effectors of the innate response modulated by MSCs, through inhibition of their respiratory burst, thus potentially limiting tissue damage (17–19); likewise, modulation of macrophage polarization promotes a switch to an anti-inflammatory phenotype (20, 21); and MSCs can also limit dendritic cell maturation and antigen presentation (22). The adaptive immune response is also importantly modulated by MSCs, through suppression of proliferation and polarization of T lymphocytes (23, 24), and through promotion of regulatory T cells (Treg) differentiation (25), all of which have an important function on immune homeostasis (26–28). In addition, MSCs can modulate the function and proliferation of B lymphocytes by promoting the generation of regulatory B cells (Breg) (29, 30). However, it is important to note that modulation of B cell function is strongly influenced by the immunological environment surrounding MSCs (31). These immunomodulatory properties have promoted research unto the use of MSCs as a therapeutic alternative for many inflammatory and immune-mediated diseases, both in humans and animals (32).

Multiple soluble mediators have been described as relevant in the inhibitory effect of MSCs on lymphocyte proliferation (33). Although precise mechanisms seem to be species-dependent, prostaglandin E_2_ (PGE_2_) secretion, indoleamine 2,3-dioxygenase (IDO) activity and nitric oxide (NO) production seem to be the most common events across species (34–37). Although there is sufficient evidence on the *in vitro* modulatory effect of MSCs on the immune response, there are disparate results between the beneficial effect of MSCs in preclinical models and their actual use in clinical diseases related to the immune system (38, 39). This discordance might be due to the unfitness of cells to survive or to operate properly in the inflammatory environment after transplantation (40). A common approach to improve MSCs survival, as well as to promote their function after arrival at the site of tissue injury, is to pre-condition MSCs *ex vivo* with different physical and chemical factors. Such factors include hypoxia, heat and oxidative stress, nutrient deprivation, or exposure to inflammatory environment mediators: TNF-α, IFN-γ, IL-1β, TLR ligands, etc. (39, 41).

In this work, we evaluated inhibition of lymphocyte proliferation by bone marrow-derived MSCs, and explored the mechanisms involved in this process. Additionally, we determined how pre-conditioning of equine MSCs with pro-inflammatory molecules modified the expression of immunomodulatory mechanisms and increased MSC's inhibition of lymphocyte proliferation.

## Materials and Methods

### Horses

Fifteen clinically healthy mixed-breed adult horses housed at Universidad Austral de Chile veterinary teaching hospital were enrolled in this study, and subdivided in lesser groups for each experiment (see below). All animals were kept on pasture, and fed with pasture grass and additional grass hay with free access to water. Physical examinations were performed by qualified veterinarians before sample collection to ensure that the animals were healthy. All procedures were approved by the Bioethics Committee for the Use of Animals in Biomedical Research of Universidad Austral de Chile, document number S55-2017.

### MSCs Isolation

BM aspirate from sternum was collected aseptically from five horses (subjects 1 to 5), four mares and one gelding, between 10 and 16 years of age as described previously (42) and slightly modified in our laboratory (19). Briefly, mononuclear cells were enriched from BM aspirates by using Percoll (Percoll^®^ GE Healthcare) gradient. The gradient was constructed putting 5 mL of BM aspirate on top of a 4 mL 70% Percoll layer into a 15 mL conical tube. After centrifugation (25 min, 400 × g), mononuclear cells were aspirated and seeded in culture flasks (T175) in low glucose (1 g/dl) Dulbecco modified Eagles minimal essential medium (DMEM, Gibco, ThermoFisher), containing 10% heat-inactivated fetal bovine serum (FBS; Biological Industries, Israel) and penicillin (100 units/mL) with streptomycin (100 μg/mL), at 37°C in an atmosphere of 5% CO_2_. After 48 h, three washes with phosphate-buffered saline (PBS) were performed in order to remove the non-adherent cells and cellular debris, while also replacing the culture medium with supplemented DMEM. The same procedure was repeated every other day, until colony forming units (CFU) were evident approximately 2 weeks after isolation. MSCs were trypsinized when cultures reached 70% of confluence, counted and cryopreserved in liquid nitrogen for subsequent experiments. MSCs were used at passage 4 or lower for all experiments described below.

### Characterization of MSCs

Molecular characterization of the MSCs was performed as described elsewhere by this study group (19). Briefly, we isolated mRNA from the resulting cell population from each of five donor horses using a commercial kit (E.Z.N.A. Total RNA kit, OMEGA bio-tek). Samples were treated with TURBO DNAse free-kit (Life Technologies) for DNA digestion. cDNAs amplification was performed using 1 μg of RNA with 200 U of M-MLV reverse Transcriptase (Promega) and 50 μM Oligo(dT)15 primer (Promega). PCRs were performed using 0.5 μM of specific primers, for CD44, CD90, CD105, CD45, and glyceraldehyde-3-phosphate dehydrogenase (GAPDH) (Table 1).

**Table 1:** Primer sequences used for MSCs culture characterization.

**Genbank code**	**Gene**	**Name**	**Sequence primer 5′→3′**	**Amplicon size**
XM_005598023.2	CD44	Homing cell adhesion molecule	F: ATCCTCACGTCCAACACCTC	165
			R: CTCGCCTTTCTTGGTGTAGC	
XM_001503225.3	CD90	Thy-1 cell surface antigen	F: TGCCTGAGCACACATACCGCTC	197
			R: GCTTATGCCCTCGCACTTGACC	
XM_003364144.3	CD105	Endoglin	F: GACGGAAAATGTGGTCAGTAATGA	101
			R: GCGAGAGGCTCTCCGTGTT	
XM_005608047.2	CD45	Protein tyrosine phosphatase receptor type C	F: TGATTCCCAGAAATGACCATGTA	101
			R: ACATTTTGGGCTTGTCCTGTAAC	

Additionally, we performed immunostaining for some surface molecules expressed by equine MSCs (43). For this purpose, we used the following antibodies: mouse anti-rat CD90 (clone OX7, Caltag Laboratories), mouse anti-human CD105 (clone SN6, Abcam, San Francisco, California, USA), and goat anti-mouse IgG (H+L)-Alexa Fluor^®^ 647 (Invitrogen) as secondary antibody. Fluorescence was evaluated using a Becton Dickinson FACS Canto II flow cytometer.

### MSCs Trilinear Differentiation

As we also described elsewhere (19), a commercial differentiation kit was used to determine the MSCs' potential trilineage differentiation (StemPro Chondrogenesis, Adipogenesis and Osteogenesis Differentiation kit, Gibco, Life Technologies). Osteogenic differentiation was confirmed by positive staining of the extracellular calcium matrix by staining with a 2% Alizarin Red S solution (Sigma-Aldrich Corp., USA), adipogenic differentiation was confirmed by the deposition of lipid droplets in the cytoplasm using 0.5% Oil Red O staining (Sigma-Aldrich Corp., USA), and chondrogenic differentiation was confirmed by staining with Alcian Blue (Sigma-Aldrich, pH = 2.5). Cells obtained from all five animals were able to differentiate into the three-cell type.

### Proliferation Assay

The effect of MSCs on lymphocyte proliferation was determined by performing a Succinimidyl Carboxyfluorescein Ester (CFSE) dilution assay, as described previously (19). Briefly, equine peripheral blood mononuclear cells (PBMC) were isolated from 10 mL of peripheral blood obtained from jugular venipuncture of 10 horses (subjects 6 to 15), using a Percoll gradient similar to the one described before. Three mL of blood were placed on a discontinuous density gradient, with 2.5 mL of 85% Percoll in the bottom of a 15 mL tube and 2.5 mL of 70% Percoll above. After centrifugation (30 min, 400 g), the upper layer containing mononuclear cells was aspirated and the cells counted. Afterwards, PBMC were loaded with Carboxyfluorescein succinimidyl ester (CFSE, Molecular probes) according to what was described in detail elsewhere (44). To induce lymphocyte proliferation, 100 μL of Roswell Park Memorial Institute (RPMI) culture medium (supplemented with FBS and antibiotics) containing 1 × 10^6^/mL of PBMC was placed per well in a 96-well culture plate and stimulated by adding 100 μL of RPMI with concanavalin A (ConA, Sigma-Aldrich Corp., USA) at 5 μg/mL, incubated for 4 days at 37°C with 5% CO_2_. Unlabeled cells with and without stimuli, and unstimulated labeled cells were used as controls of the technique. Dilution of the CFSE label was evaluated by flow cytometry, for which the cells were transferred to cytometry tubes, washed and labeled with LIVE / DEAD^®^ (FixableNear-IR Dead Cell Stain, ThermoFisher Scientific), according to the manufacturer's instructions. Once the incubation was finished, the cells were washed 2 times with 1 mL of cytometry buffer, resuspended in 200 μL and evaluated by flow cytometry.

### MSCs on Lymphocyte Proliferation

PBMC and MSCs were co-cultured in different ratios (PBMC:MSC of 1:1, 4:1, 16:1, 32:1, and 64: 1, respectively). For this purpose, a variable number of MSCs were cultured in DMEM medium with 10% of fetal bovine serum and 24 h later, each well was washed with PBS and freshly isolated 1 × 10^5^ PBMC resuspended in RPMI were added. Subsequently, the dilution of the CFSE was determined, in order to calculate the percentage of proliferation after stimulation with ConA (5 μg/mL). To determine the mechanisms through which equine MSCs inhibit lymphocyte proliferation, relevant molecules proposed as responsible for this effect were pharmacologically inhibited. PGE_2_ production was inhibited by the addition of indomethacin (Sigma-Aldrich) to the co-culture of MSCs and PBMC. IDO activity was inhibited by using 1-Methyl-D-tryptophan (1-MT, Sigma-Aldrich). Finally, the inducible form of nitric oxide synthetase (iNOS) was inhibited using Aminoguanidine (AG, Sigma-Aldrich). All pharmacological inhibitors were tested at different concentrations; this study's results list only the maximum concentration that does not affect lymphocyte proliferation. Chosen concentrations were similar to those previously used elsewhere (45, 46), and close to the described IC50 for each inhibitor (14 uM for indomethacin, 7 uM for 1-MT and 2.1 uM for AG) (47–49).

To determine the effect of pre-conditioning with TNF-α, IFN-γ, or their combination on the inhibitory effect of MSCs on PBMC proliferation, a co-culture assay was performed as was described above. Briefly, a ratio 16:1 was used, 15,625 MSCs were seeded in a 24-well plate (per duplicate) in DMEM containing 10 ng/mL of TNF-α or IFN-γ during 24 h at 37°C with 5% CO_2_. Thereafter, MSCs were washed twice with PBS and 2 × 10 PBMC unlabeled or labeled with CFSE were seeded (ratio PBMC 16:1) with or without 5 μg/mL of ConA as unspecific stimuli. Dilution of the CFSE label was evaluated by flow cytometry (BD FACS Canto II). Data are presented as percentage of proliferation and proliferation index. The former is the percentage of events within the lymphocyte gate that show a dilution of fluorescence, which indicates cell division. The proliferation index was used to take into account individual variation in baseline lymphocyte proliferation rates within PBMCs by standardizing treatments for each horse to the lymphocyte proliferation rates in PBMC without added MSC for that horse. The proliferation rates in PBMC without added MSC was set at 1.0.

### Pre-conditioning of MSCs Cultures

For determination of the pre-conditioning effect with pro-inflammatory molecules on expression of immunomodulatory genes by MSCs, 3x10^5^ MSCs were seeded in a 6-well culture plate in DMEM culture medium, supplemented with 10% of FBS and antibiotics as described above. The next day, the culture medium was replaced with DMEM medium containing different concentrations (0.1, 1, or 10 ng/mL) of equine TNF-α, IFN-γ, or their combination (R&D System) during 24 h at 37°C with 5% CO_2_, after which cells were lysed and RNA isolated as is described below.

### RNA Isolation, cDNA Synthesis and PCR

RNA isolation was performed using E.Z.N.A Total RNA Kit I (Omega), following the manufacturer's instructions. Genomic DNA was removed with the kit DNAse Turbo (Thermofisher), after which RNA was checked for integrity, quantified by nanodrop and stored at −80°C. One μg of total RNA from each sample was used for cDNA synthesis using oligonucleotides and M-MLV reverse transcriptase (Promega), following the manufacturer's instructions. Primers used for interleukin 6 (IL-6), Prostaglandin-endoperoxide synthase 2 (PTGS2, formerly known as COX-2), IDO, iNOS, hepatocyte growth factor (HGF), and transforming growth factor β (TGF-β), are listed in Table 2. Levels of gene expression were obtained using the comparative 2^−ΔΔ*Ct*^ method described elsewhere (50), and as housekeeping gene, Ribosomal Protein L32 (RPL32) was used. All reactions were carried out in triplicate and using Fast SYBR Green Master Mix. RT-qPCR reactions were performed and monitored using a StepOne Real Time PCR System device (ThermoFisher, Scientific).

**Table 2:** Primer sequences used to evaluate the effect of pre-conditioning on the expression of immunomodulatory molecules by MSCs.

**Genbank code**	**Gene**	**Name**	**Primer secuence 5′→3′**	**Amplicon size**
NM_001081775.2	PTGS2	Prostaglandin-endoperoxide synthase 2	F: GATCCTAAGCGAGGTCCAGC	101
			R: AGGCGCAGTTTATGCTGTCT	
XM_014739139.2	HGF	Hepatocyte growth factor	F: TGCATTCAAGGTCAAGGAGA	234
			R: TTTTGGAATTTGGGAGCAGT	
XM_014736538.2, XM_014736539.2	IDO	Indoleamine 2, 3 dioxygenase	F: ACAACATCAGGACCAGGACAC	197
			R: TCCAGACGCCTTCATAGAG	
NM_001082496.2	IL-6	Interleukin-6	F:ATGAGTGGCTGAAGAACACAACAAC	131
			R:AGGAATGCCCATGAACTACAACAAT	
NM_001081769.1	iNOS	Inducible nitric oxide synthase	F: CCAACAATGGCAACATCAGGT	85
			R: TGAGCATTCCAGATCCGGA	
XM_005596086.2	TGF-β	Transforming growth factor beta	F: GGAATGGCTGTCCTTTGATG	120
			R: CGGAGTGTGTTATCTTTGCTGTC	
XM_001492042.6	RPL-32	Ribosomal protein L32	F: GGAATGGCTGTCCTTTGATC	138
			R: CGGAGTGTGTTATCTTTGCTGTC	

### Data Analysis

GraphPad Prism (GraphPad Software Inc., version 8.0) was used for graph generation and statistical analysis. Shapiro Wilks tests showed that the data were not normally distributed for all expression and proliferation experiments. Kruskal-Wallis test was performed to compare the differences between groups in each assay, and this was followed by a Tukey test. Overall, *p* < 0.05 were considered as significant.

## Results

### Characterization of Equine Bone Marrow-Derived MSCs

**Figure 1:**
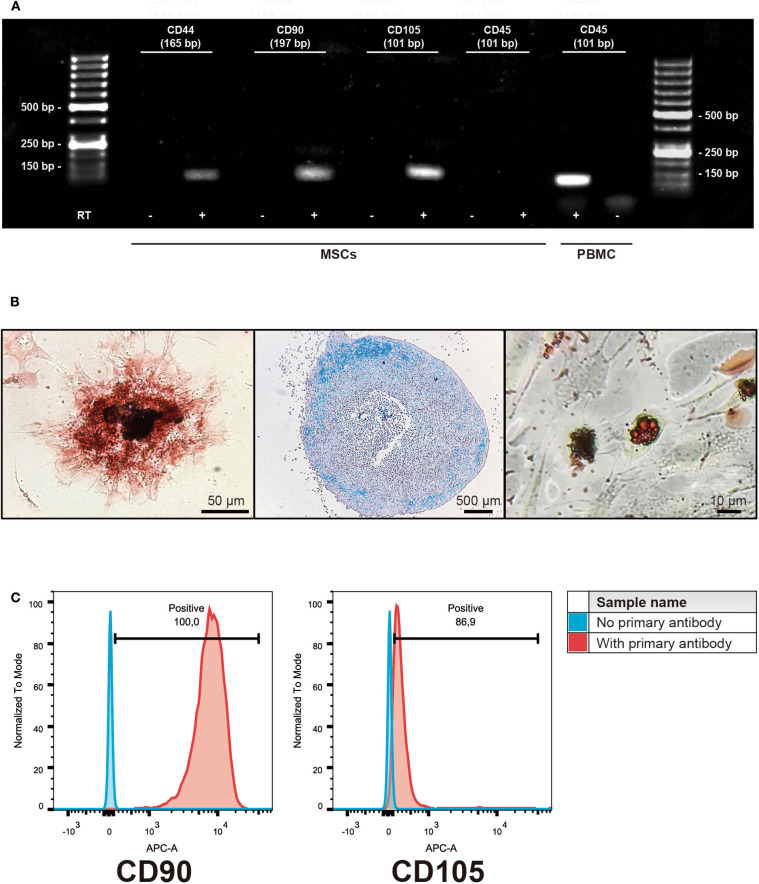
Characterization of isolated bone marrow-derived mesenchymal stromal cells (MSCs). **(A)** Representative gel of the isolated subpopulations, indicating the expression of MSCs markers CD44, CD90, and CD105; hematopoietic marker CD45 expression was absent in MSCs. PBMC cDNA was used as a control of CD45 expression. (+) indicates cDNA synthesis including reverse transcriptase (RT) while (-) indicates controls without the enzyme in order to identify amplification of non-specific products. **(B)** Representative example of trilinear differentiation of isolated bone marrow-derived MSCs, showing the osteogenic lineage stained with Alizarin red 2% (left panel), chondrogenic stained with Alcian blue 1% (middle panel) and adipogenic stained with Oil red (right panel) lineages. **(C)** Representative histograms of flow cytometry determinations for CD90 and CD105 of the isolated cell populations.

Equine MSCs were obtained by culturing the bone marrow-derived mononuclear cells obtained after the gradient centrifugation. After 2 or 3 weeks of culture, cells reached 70% of confluence and were detached and cryopreserved in liquid nitrogen. The cells obtained were negative for the hematopoietic linage marker CD45 but they did express CD44, CD90, and CD105. This was also confirmed by flow cytometry, showing expression of CD90 (mean ± standard deviation 99.4 ± 0.2%) and CD105 (88 ± 4.6%) in the isolated populations. Additionally, the cells were successfully induced to differentiate into osteocyte, chondrocyte and adipocyte lineages (Figure 1).

### Inhibition of Lymphocyte Proliferation by Equine Bone Marrow-Derived MSCs

**Figure 2:**
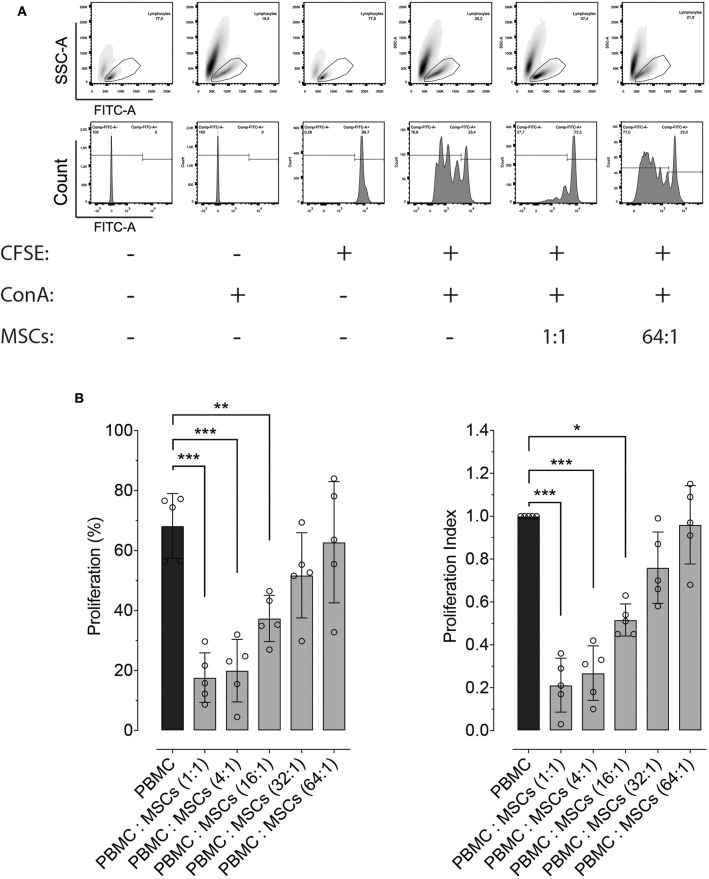
Effect of co-culture with MSCs on lymphocyte proliferation. **(A)** Representative flow cytometry images showing the effect of co-culture of peripheral blood mononuclear cells (PBMC) and two distinct ratios of MSCs, on the proliferation of lymphocytes. The top panels show the gating strategy to assess lymphocyte proliferation within PBMCs, while the lower panel represents the fluorescence intensity of CFSE on the gated lymphocytes. Proliferation was induced by incubation with 5 μg/mL of concanavalin A (ConA) and determined by measuring CFSE dilution. **(B)** Effect of co-culture between PBMC and MSCs at different ratios on lymphocyte proliferation (left panel), and the same results normalized by the lymphocyte proliferation within PBMCs without MSCs (set at 1.0, black bar). (^*^p < 0.05, ^**^p < 0.01, ^***^p < 0.001; *n* = 5).

Besides molecular expression and potency, inhibition of lymphocyte proliferation is a hallmark ability of MSCs, independently of the tissue from which they were isolated. Here we demonstrate that equine bone marrow-derived MSCs inhibit lymphocyte proliferation after being induced with non-specific mitogenic stimuli. Inhibition of lymphocyte proliferation was determined by co-culturing PBMC and MSCs at different ratios, which showed that MSCs were capable of inhibiting lymphocyte proliferation up to a 16:1 ratio (Figure 2).

### Mechanisms of MSCs-Mediated Inhibition of Lymphocyte Proliferation

**Figure 3:**
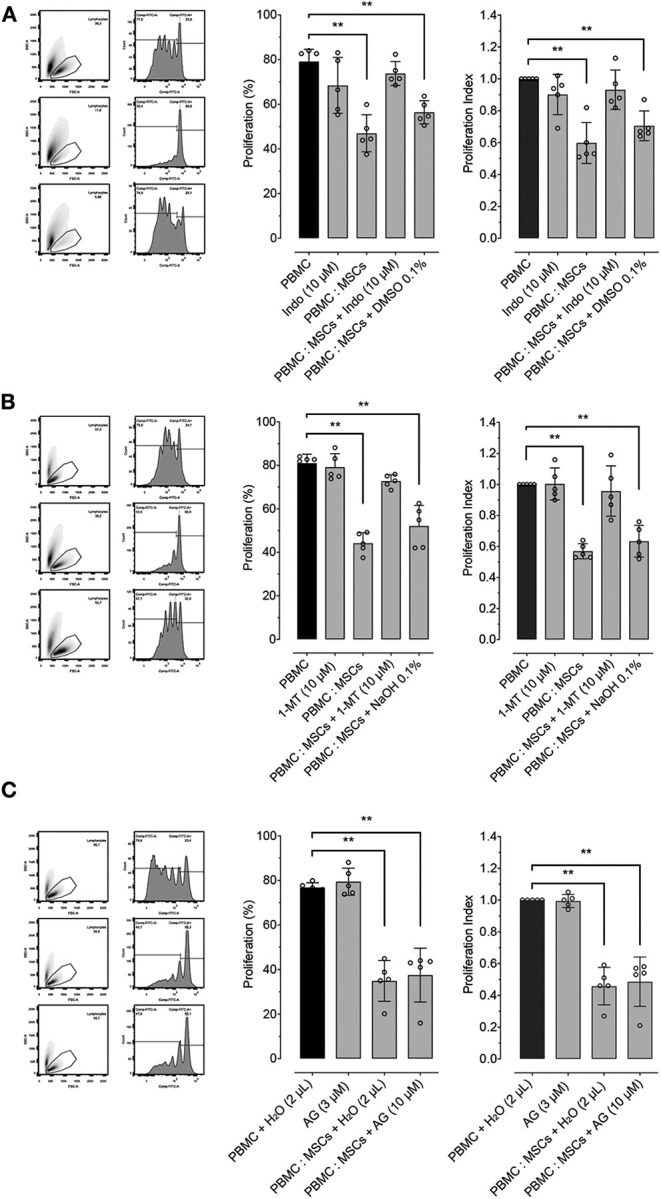
MSCs inhibitory effect on lymphocyte proliferation is dependent on PTGS2 and IDO function. Lymphocyte proliferation induced by incubation of peripheral blood mononuclear cells (PBMC) with 5 μg/mL of concanavalin A (ConA), cultured alone or co-cultured with MSCs at 16:1 ratio. **(A)** Effect of prostaglandin-endoperoxide synthase 2 (PTGS2) inhibition with 10 μM of indomethacin on the inhibitory effect of MSCs on lymphocyte proliferation. **(B)** Effect of indoleamine 2, 3 dioxigenase (IDO) inhibition with 2 μM of 1-Methyltryptophan (1-MT) on the inhibitory effect of MSCs on lymphocyte proliferation. **(C)** Effect of inducible nitric oxide synthase (iNOS) inhibition with 1.5 μM of aminoguanidine (AG) on the inhibitory effect of MSCs on lymphocyte proliferation. Two μL of water was added to the control condition as vehicle (^**^p < 0.01; *n* = 5).

In order to determine the role of important molecular pathways commonly involved in the inhibitory effect of MSCs on lymphocyte proliferation in different species, we explored the role of PGE_2_, IDO and iNOS by using pharmacological inhibition of these molecules during PBMC: MSCs co-culture. PGE_2_ synthesis was inhibited using indomethacin (a COX inhibitor). Indomethacin reverted MSCs' inhibition of PBMC proliferation, while its vehicle does not have a significant inhibitory effect (Figure 3A). For exploration of the role of the IDO pathway on the effect of MSCs on proliferation, we used 1-MT as an IDO inhibitor. We demonstrated that IDO does participate in the inhibitory effect of MSCs (Figure 3B). Lastly, we tested if iNOS activity was related with MSCs function by using AG (Figure 3C), showing that AG produced no significant reversion of the inhibitory effect of MSCs on PBMC proliferation.

### Pre-conditioning Effect in the Expression of Immunomodulatory Molecules by MSCs

**Figure 4:**
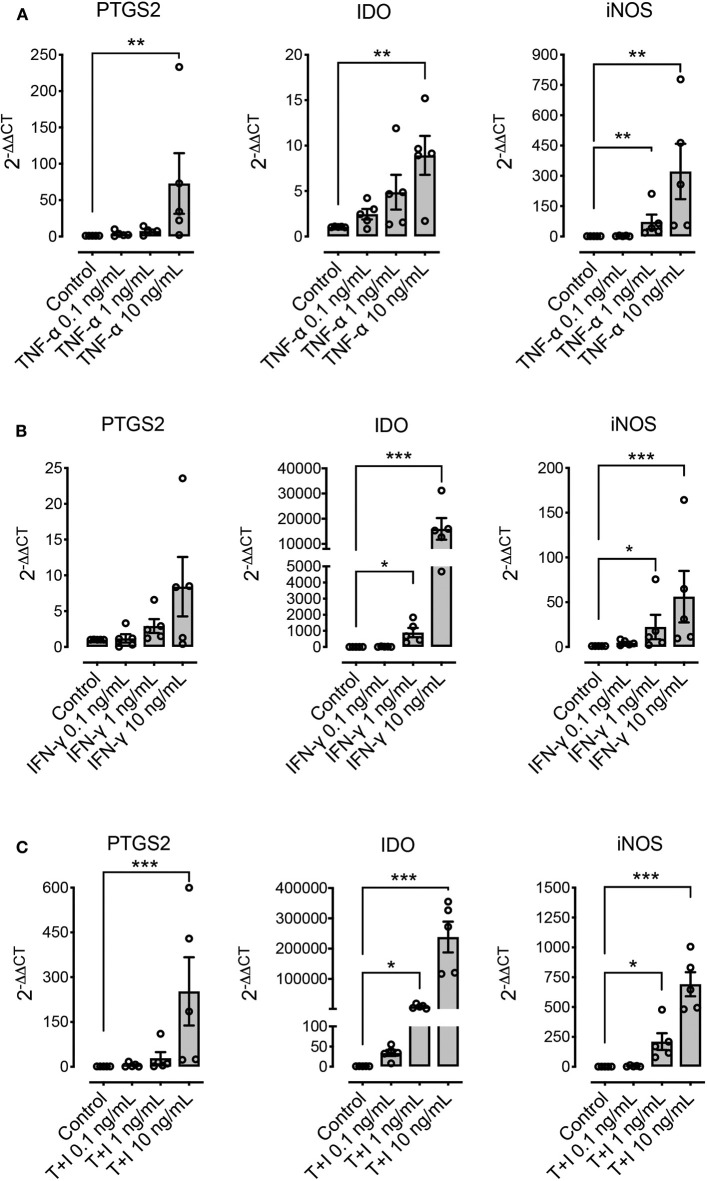
Pre-conditioning increases the expression of immunomodulatory genes in MSCs. The effect of pre-conditioning equine bone marrow-derived MSCs during 24 h with TNF-α **(A)**, IFN-γ **(B)** or its combination **(C)** on the expression of prostaglandin-endoperoxide synthase 2 (PTGS2), indoleamine 2, 3 dioxigenase (IDO), and inducible nitric oxide synthase (iNOS) (^*^p < 0.05, ^**^p < 0.01, ^***^p < 0.001; *n* = 5).

We explored the effect of pre-conditioning MSCs with pro-inflammatory molecules TNF-α, IFN-γ, or their combination, on the expression of immunomodulatory molecules defined as relevant in other species: PTGS2, IDO, and iNOS. Pre-conditioning with TNF-α showed a significant increase in the expression of PTGS2, IDO and iNOS (Figure 4A). Pre-conditioning with IFN-γ produced a significant upregulation of iNOS and specially IDO, while PTGS2 showed no change in its expression (Figure 4B). Combined pre-conditiong of MSCs with both TNF-α and IFN-γ produced a significant upregulation of PTGS2, IDO, and iNOS, especially at the higher concentration (Figure 4C).

**Figure 5:**
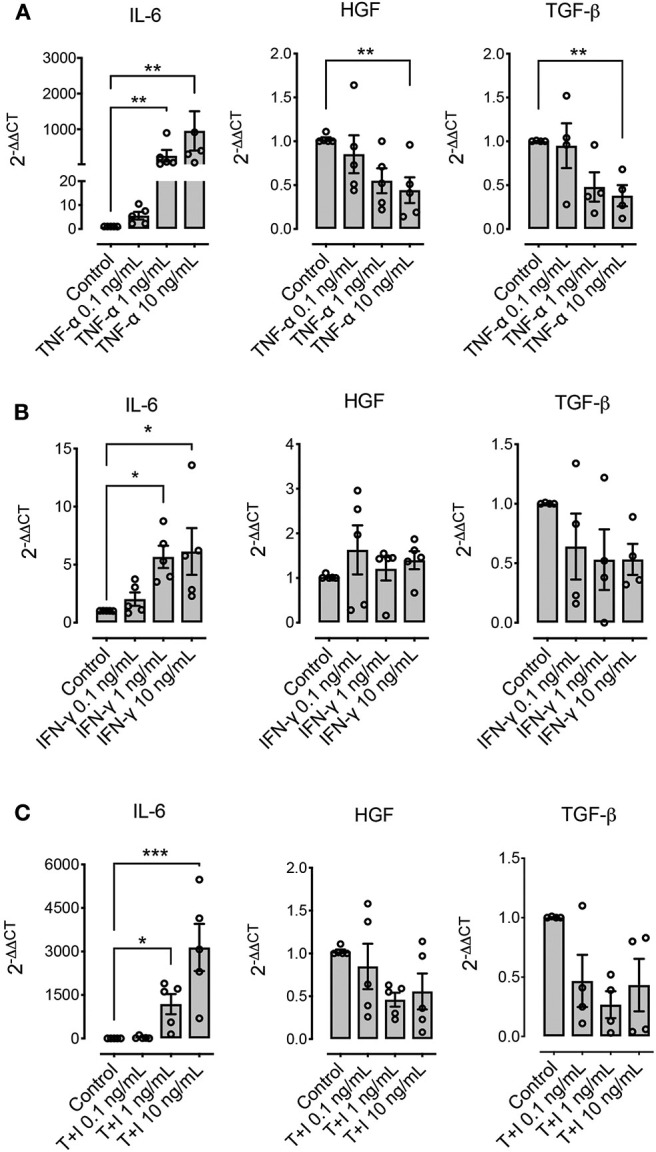
Pre-conditioning modulates the expression of immunomodulatory genes in MSCs. The effect of pre-conditioning equine bone marrow-derived MSCs during 24 h with TNF-α **(A)**, IFN-γ **(B)** or their combination **(C)** on the expression of interleukin 6 (IL-6), hepatocyte growth factor (HGF), and transforming growth factor β (TGF-β) (^*^p < 0.05, ^**^p < 0.01, ^***^p < 0.001; *n* = 4 or 5 since we were unable to determine the basal expression of TGF-β in one animal).

Other molecules that have been described as relevant immunomodulatory mediators in other species are IL-6, HGF, and TGF-β. Pre-conditioning with TNF-α, IFN-γ, or their combination significantly increased the expression of IL-6, particularly when TNF-α was used as stimuli (Figure 5). On the other hand, HGF and TGF-β showed a downregulation at the highest concentrations when TNF-α was used alone, and no change in their expression levels was shown when IFN-γ was used alone or in combination with TNF-α (Figure 5).

### Pre-conditioning Increases the Inhibitory Effect of MSCs on PBMC Proliferation

**Figure 6:**
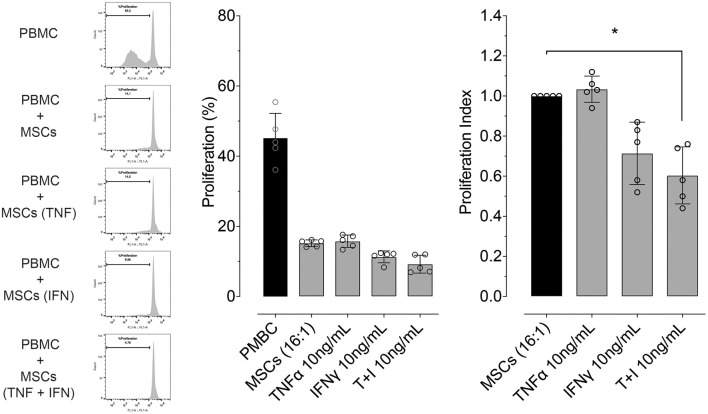
Pre-conditioning increases the inhibitory effect of MSCs on lymphocyte proliferation. Left: representative flow cytometry images showing the inhibitory effect on lymphocyte proliferation, of a co-culture between peripheral blood mononuclear cells (PBMC) and MSCs in different conditions (naïve or preconditioned with TNF-α, IFN-γ or their combination). Proliferation was induced by incubation with 5 μg/mL of concanavalin A (ConA) and determined by CFSE dilution. Right: inhibitory effect on lymphocyte proliferation of a co-culture between PBMC and MSCs in different conditions (naïve or preconditioned with TNF-α, IFN-γ or their combination) (^*^p < 0.05; *n* = 5).

We determined whether pre-conditioning MSCs with pro-inflammatory molecules TNF-α, IFN-γ modulates their inhibitory effect on PBMC proliferation or not. Since the maximum upregulating effect on gene expression of PTGS2 and IDO was obtained with the highest concentration of TNF-α and IFN-γ (10 ng/mL), this same TNF-α and IFN-γ concentration was used to evaluate MSC-mediated inhibition of lymphocyte proliferation. We did not observe an effect of pre-conditioning with TNF-α on inhibition of lymphocyte proliferation, priming MSCs with IFN-γ show an increase in this capacity not showing statistical significance. However, pre-conditioning of MSCs with a combination of both TNF-α and IFN-γ did significantly increase MSCs-mediated inhibition of lymphocyte proliferation (Figure 6).

## Discussion

The immunomodulatory potential of MSCs is one of their most important characteristics, giving them therapeutic potential for a number of immune-mediated pathologies. It is already known that MSCs can inhibit lymphocyte proliferation, regardless of the species or the site of obtention (11, 51–55). Lymphocytes, and particularly T cells, are one of the main effector cells of the adaptive immune response, mastering the type of response against a specific pathogen (56). For this process, clonal expansion upon stimulation is key to mounting a successful immune response (57). However, this process must be regulated in such way that will be effective at eliminating the infection, without triggering an overwhelming inflammatory response such as occurs in allergies or auto-immune diseases. Likewise, uncontrolled inflammation can perpetuate or worsen infectious diseases, such as sepsis and its multisystemic deleterious consequences (58). Thus, MSCs are of definite interest as immune modulators or anti-inflammatories (59). The suppression of lymphocyte proliferation by MSCs has been shown to be mediated by different mechanisms that vary among species (34). In humans the expression by MSCs of IDO, PGE_2_, HGF, TGF-β, IL-6, human leukocyte antigen-G (HLA-G), tumor necrosis factor-α-stimulated gene/protein 6 (TSG-6), among other molecules, seems to command their suppressive effect (33). iNOS is essential in mice for the inhibition of T cell proliferation by MSCs, whose expression is strongly induced by IFN-γ in combination with other inflammatory cytokines (55).

Either autologous or allogeneic equine MSCs have also shown a strong inhibitory effect on lymphocyte proliferation (11, 46, 60). Our approach was to test the *in vitro* capacity of allogeneic MSCs to inhibit lymphocyte proliferation, observing a strong inhibition depending on the ratio between both cell populations. The only molecule described as involved is PGE_2_, while IDO and iNOS do not seem to be involved in this immunomodulatory process (46). Similar to what was described earlier for equine BM-derived MSCs, our results show that cyclooxygenase (COX) pharmacological inhibition restores the proliferation of lymphocytes in co-culture with MSCs (45, 46). Since PGE_2_ was identified as a molecule secreted in the culture media of human MSCs (61), it has been described as a major mediator of MSCs modulatory effects in several species (11, 37, 53, 62), not only inhibiting lymphocyte proliferation, but also inhibiting the expression of co-stimulatory molecules by antigen presenting cells (APCs), thus limiting their capacity to activate T cells. Additionally, MSCs produce PGE_2_ in sufficient quantity to be able to reprogram macrophages, thus inhibiting production of TNF-α and IL-6 and increasing the secretion of IL-10 (63, 64). iNOS-NO has also been shown to be a fundamental axis for murine MSCs-mediated inhibition of lymphocyte proliferation (34, 55), but this has not be seen in other animal species or humans. Here, we found that iNOS inhibition does not change the inhibitory effect of equine MSCs on lymphocyte proliferation, similar to what was described earlier for equine MSCs (45, 46).

The enzyme IDO is another relevant immunomodulatory molecule expressed by MSCs, and seems to be an important pathway for human MSCs' inhibitory effect on lymphocyte proliferation (65). The role of IDO as an immunomodulator was first described in professional antigen presenting cells (APCs), in whom expression of IDO induced by IFN-γ and other proinflammatory cytokines catalyzed conversion from tryptophan to kynurenine, inducing a metabolic disruption and acting as a major immunosuppressive effector pathway that inhibits T-cell responses (66). Expression of this molecule is a conserved mechanism associated to the immunoregulatory role of other cells such as regulatory T cells (67). Unlike what is described in the literature for equine MSCs (45, 46), here we show that IDO activity is also essential for maintaining their inhibitory effect on lymphocyte proliferation, which is consistent with the role of this enzyme in the modulatory effect of MSCs described in other species such as humans, pigs and cats (68, 69). The differences and similarities between different species could be partially explained by their phylogenetic relationship (24).

Since MSCs immunosuppressive functions require preliminary activation by immune cells through the secretion of pro-inflammatory cytokines (55), the pre-conditioning or “priming” of MSCs with different molecules prepare the cell to initiate the mechanisms involved in the modulation of the immune response and potentially increase MSCs' immunomodulatory effect. Here we show that incubation of MSCs with TNF-α, IFN-γ or their combination effectively increases the expression of immunomodulatory molecules such us PTGS2 and IDO, which participate in the inhibition of lymphocyte proliferation carried out by the MSCs. This has been demonstrated before in MSCs obtained from other species, where MSCs exposure to TNF-α and/or IFN-γ increase their modulatory properties over lymphocytes and other immune cells (70–72). In horses, a study described that pre-conditioning with a combination of these pro-inflammatory molecules induced an upregulation of iNOS and IDO, while PTGS2 expression did not change (73). This divergence could be due to the differences in the time of stimulation, since the previous study preconditioned the cells for only 12 h and with half of the maximum concentration used in the present work, which may not have been enough to induce a significant response. However, the great variability shown by the expression of this molecule in response to stimulation in both studies, could indicate an important degree of individual response to stimulation.

Other molecules described as relevant for the immunomodulatory effect of MSCs, such as HGF and TGF-β, show no changes in their expression after pre-conditioning with IFN-γ or the combination of both cytokines, or a down-regulation in their expression after pre-conditioning with TNF-α, which is similar to the response seen for murine MSCs for TGF-β, but not for the expression of HGF (70). This result is also in line with the results from for equine BM-derived MSCs, where pre-conditioning with a combination of both pro-inflammatory cytokines induces a down regulation of TGF-β and IL-10 (73).

Herein we show that pre-conditioning of MSCs with pro-inflammatory mediators (TNF-α and IFN-γ) produced a joint increase in gene expression of regulatory mediators and in lymphocyte proliferation inhibitory potential. This coincides with reports on human BM-derived MSCs (71, 74, 75), in which PBMCs stimulated with PHA and co-cultured with MSCs pre-conditioned with IFN-γ showed diminished proliferation. **I**n the present work lymphocytes co-cultured with MSCs pre-conditioned with IFN-γ showed no significant decrease in their proliferation despite the fact that there is a trend toward this effect; more replicates may have been informative of the effect. Thus, such stimuli seem to be relevant when assessing MSCs' lymphocyte proliferation inhibitory potential. Since we used PBMC for these experiments rather than pure lymphocyte cultures, we cannot absolutely rule out that the inhibitory effect on lymphocyte proliferation could also be mediated by other cells present in the culture, such as monocytes; in this scenario, the immune response might have been altered through the secretion of pro-inflammatory cytokines and suppressive cytokines like IL-10 by other cell types.

Regarding interaction between MSCs and immune system, particularly in an allogeneic approach as the present work, the pre-conditioning treatment could induce an increase in their immunogenicity and therefore a loss of their modulatory effect on inflammation. In line with this concern, Barrachina et al. (73), showed that the priming of equine BM-derived MSCs with a combination of TNF-α and IFN-γ induces an upregulation of major histocompatibility molecules (MHC-II) and a co-stimulatory molecule (CD40) 12 h after treatment. This is in line to what is described for humans, where both cytokines seem to induce a loss of the hypoimmunogenic condition of the MSC, since IFN-γ induces a strong upregulation of MHC-I, MHC-II and co-stimulatory molecules in comparison with the TNF-α treatment (76). However, this increase in the immunogenicity of MSCs does not seem to translate completely into a loss of their *in vivo* immunomodulatory effect. The same authors tested preconditioned cells with the TNF-α and IFN-γ combination on the ability of MSCs to modulate joint inflammation, and although they reported an increased reactive effect in a second administration of the MSCs, this was self-limiting and resolved without the need for additional medication (77).

In conclusion, results show that equine BM-derived MSCs effectively inhibit lymphocyte proliferation in a PTGS2 and IDO dependent manner, and that pre-conditioning with proinflammatory cytokines (TNF-α and IFN-γ) sets in motion a number of key immunomodulatory mechanisms increasing their inhibitory effect on lymphocyte proliferation. Although the main limitation of this type of study is the low number of subjects used sample collection, the evidence obtained warrants further research into the immunomodulatory potential of MSCs.

## Data Availability Statement

The raw data supporting the conclusions of this article will be made available by the authors, without undue reservation, to any qualified researcher.

## Ethics Statement

The animal study was reviewed and approved (resolution number 268/2016) by Bioethics Committee for the Use of Animals in Biomedical Research of Universidad Austral de Chile.

## Author Contributions

VC obtained and processed the biological samples and performed the majority of the experimental procedures. GE perform experiments related with the pharmacological inhibition in order to determine the mechanisms involved in the inhibitory effect of MSCs. GG and NM took part in the bone marrow sampling and isolation of the mesenchymal stem cells. BU, MD, and GM helped to draft the manuscript and with data interpretation. AP participated in the experimental design and performed MSCs isolation and characterization, and together with CH participated in the design of the study, sample collection, and processing.

## Conflict of Interest

The authors declare that the research was conducted in the absence of any commercial or financial relationships that could be construed as a potential conflict of interest.
